# Emergency Peripartum Hysterectomy During COVID-19 Pandemic

**DOI:** 10.7759/cureus.20524

**Published:** 2021-12-20

**Authors:** Rajlaxmi Mundhra, Anupama Bahadur, Shloka Sharma, Dipesh K Gupta, Mahima Mahamood M, Purvashi Kumari, Rabia Zaman, Pranoy Paul, Shalinee Rao

**Affiliations:** 1 Obstetrics and Gynecology, All India Institute of Medical Sciences (AIIMS) Rishikesh, Rishikesh, IND; 2 Division of Molecular Biology, Proteomics and Metabolomics, All India Institute of Medical Sciences (AIIMS) Rishikesh, Rishikesh, IND; 3 Pathology and Laboratory Medicine, All India Institute of Medical Sciences (AIIMS) Rishikesh, Rishikesh, IND; 4 Pathology, All India Institute of Medical Sciences (AIIMS) Rishikesh, Rishikesh, IND

**Keywords:** postpartum haemorrhage, obstetric hysterectomy, morbidly adhered placenta, emergency peripartum hysterectomy, caesarean hysterectomy

## Abstract

Objective

To evaluate women undergoing emergency peripartum hysterectomy (EPH) during COVID-19 pandemic regarding their sociodemographic features, indications, intraoperative and postoperative complications, and assess their health problems related to a traumatic birth.

Methods

This was a retrospective review of EPH cases operated from March 2020 to March 2021 in terms of demographic characteristics, intraoperative, and postoperative outcome variables.

Results

During the specified time period, there were nine cases of EPH. All patients were young with ages ranging from 25 to 31 years; all were unbooked having unplanned pregnancies and presented at varying gestational ages. Six out of nine cases (66.67%) had previously scarred uterus with five women having morbidly adhered placenta. A total of 77.78% (seven out of nine) patients referred to our centre with high-risk factors. Five out of nine women (55.56%) needed ICU care. Seven out of nine women (77.78%) had live births and two of these infants died. The guilt of losing the baby, lethargy, worries related to feminity and sexual health, and flashbacks of ICU stay were major concerns.

Conclusion

The morbidly adhered placenta was the primary cause of EPH in our study cohort. Preventive psychological session should be an integral part of postpartum follow-up visits for any women with traumatic childbirth.

## Introduction

The COVID-19 pandemic has grossly affected the lives of people throughout the world [1]. The Indian Government announced a nationwide lockdown in the initial phase starting from 23rd March 2020 as a preventive step to minimize its spread [2]. Major changes were made in various Government policies especially involving the health system. Though health facilities continued to provide their essential services, elective surgeries had to be curtailed to divert the hospital resources in managing the COVID-19 pandemic. In association with FOGSI (Federation of Obstetricians and Gynaecologists of India), the Ministry of Health and Family Welfare issued guidelines to provide uninterrupted health facilities to pregnant women considering them to be a high-risk group [3]. Despite this, evidence suggested a sharp decline in acceptability of essential maternal health care during the strict lockdown [4]. Restrictions on travel, fear of being exposed to COVID-19 infection, and limited health facilities in low resource areas further minimized antenatal visits. This neglected health check-up status resulted in many women attending labour stages with high-risk factors, making them prone to labour complications and emergency surgery.

Labour and childbirth induce an array of emotions in the parturient ranging from joy and happiness to pain and grief in the event of a traumatic birth [5]. Every woman enters the delivery room with the hope of experiencing joy and pride with her motherly instinct [6]. But at times, this joyous event can result in a negative emotional experience in sadness, emptiness, and a sense of worthlessness [7-9]. Emergency peripartum hysterectomy (EPH) is one such life event wherein the parturient can severely affect her physical, emotional, and psychological health. Literature suggests that one in 1000 women globally have EPH [10,11]. Little is known about the emotional and psychological effects of caesarean hysterectomy. The patient population herein is a young woman, already in a fragile state of mind, and subjecting her to this traumatic life event can harm her state of mind. Traumatic childbirth experience is known to be associated with posttraumatic stress disorder (PTSD) [12].

Moreover, the present pandemic scenario may also affect her emotional and social functioning. With the emergence of the COVID-19 pandemic, every new patient in our hospital is a COVID suspect unless the report comes out negative. Hence, pregnant women needing immediate delivery without her COVID test reports undergo necessary care in COVID suspect area with an undertaking of being getting COVID infection from other patients in the same room if any of them comes positive for infection. This itself adds to her stress. With this aim in mind, we planned to evaluate women undergoing EPH during the COVID-19 pandemic regarding their sociodemographic features, indications, intraoperative and postoperative complications, and assess their health problems related to a traumatic birth. This review is unique because it is the first article reviewing the details of EPH cases with a focus on their self-related health issues related to a traumatic birth.

## Materials and methods

This was a retrospective review of EPH cases conducted in the Department of Obstetrics and Gynaecology at a tertiary care centre from Uttarakhand, India. Being a referral centre, we receive many high-risk patients in critical condition. From March 2020 to March 2021, we encountered nine cases of emergency peripartum hysterectomy. This study was approved by the Institutional Ethical Committee of All India Institute of Medical Sciences, Rishikesh vide letter number AIIMS/IEC/21/405. COVID-19 pandemic badly affected health systems all over the world and our institute was also one of them. All elective facilities were shut and visits were mostly restricted for emergency needs. This institute caters to the hilly population and nationwide lockdown resulted in difficult transportation even at times of emergency. We started telemedicine facilities to continue providing routine antenatal care thereby restricting routine physical visits for low-risk women. Emergency services were running taking all COVID precautions. For every unbooked case or referred case, the reason for delay was noted. For cases undergoing EPH, we evaluated the case files from hospital records and noted the number of antenatal visits, a referral from other centre, sociodemographic parameters such as age, parity, period of gestation, planned/unplanned pregnancy status, presenting complaints, reasons for the delay in seeking medical help, imaging findings, mode of delivery, and operative variables like indication for EPH, type of uterine incision, intraoperative findings, blood loss, number of blood and blood components transfused, maternal ICU admission, and duration of hospital stay. Infant characteristics in terms of live birth, birth weight, neonatal ICU admission, and period of separation from baby were all recorded in the proforma. Details regarding their self-reported health issues related to traumatic birth were also recorded from their case files (when they came for follow-up visit at six weeks).

Statistical analysis

Baseline demographic characteristics, intraoperative, and postoperative outcome variables of all cases of EPH were noted in a tabular form. Descriptive statistics were used to calculate simple frequency, percentage, and proportion.

## Results

During the specified period, we had nine cases of emergency peripartum hysterectomy. Table 1 shows the baseline demographic characteristics of the study sample with reasons for the delay in seeking a health care facility. All patients were young, with ages ranging from 25 to 31 years. All of these were unbooked having unplanned pregnancies and presented at varying gestational ages. Six out of nine cases (66.67%) had previously scarred uterus. Case number 8 presented at day 2 of puerperium with placenta accreta and postpartum hemorrhage with puerperal sepsis. Seven out of nine cases (77.78%) were referred cases. One of the women was referred with COVID positive status.

**Table 1:** Baseline characteristics of study participants GPAL: Gravida Parity Abortion Live birth; PPH: Postpartum haemorrhage; APH: Antepartum haemorrhage; USG: Ultrasonography; LSCS: Lower segment caesarean section; NICU: Neonatal intensive care unit.

S. no	Age	GPAL status	Booking status	Gestational age on admission in current pregnancy	Pregnancy intention (planned or unplanned)	Mode of delivery	History of mental illness	Type of family (nuclear/joint)	Reasons for delay in seeking health care
1	25	G3P2L1 Previous two caesarean	unbooked	37 weeks 1 day	unplanned	caesarean	Nil	Nuclear	Fear of COVID infection, transport problem
2	30	G4P3L3 previous three caesareans	unbooked	35 weeks 6 days	unplanned	caesarean	nil	nuclear	Got referred from local hospital due to non-availability of anaesthetist
3	30	G3P2L2 previous two caesareans	unbooked	28 weeks 5 days	Unplanned	Caesarean	Nil	nuclear	Referred with APH and a USG report of central placenta praevia, percreta, anhydramnios and fetal multicystic kidneys
4	29	G4P2L2A1, previous two caesarean	unbooked	40+2 weeks	unplanned	Caesarean	Nil	Nuclear	Referred with COVID positive status
5	30	G3P2L2, previous two vaginal deliveries	Unbooked	40+1 week	Unplanned	Caesarean	Nil	Nuclear	Referred from a local hospital in view of Meconium-stained liquor and non-availability of NICU facility
6	27	G4P2l2A1 with previous one caesarean	Unbooked	23+3 weeks	Unplanned	Caesarean	Nil	Nuclear	Referred with bleeding per vaginum and frank haematuria and USG showing placenta percreta
7	29	G2P1L1 with previous preterm LSCS	Unbooked	16+5 weeks	Unplanned	Caesarean	Nil	Nuclear	Fear of COVID infection
8	31	P2l1A1 at day 2 of caesarean section (done in view of placenta accrete, placenta left in situ)	Unbooked	Day 22 of LSCS	Unplanned	Caesarean	Nil	Nuclear	Referred in view of placenta accreta and PPH with puerperal sepsis
9	25	P1L0 at day 1 of vaginal delivery	Unbooked	Day 1	Unplanned	Vaginal delivery	Nil	Nuclear	Referred in view of PPH in haemorrhagic shock

**Figure 1:**
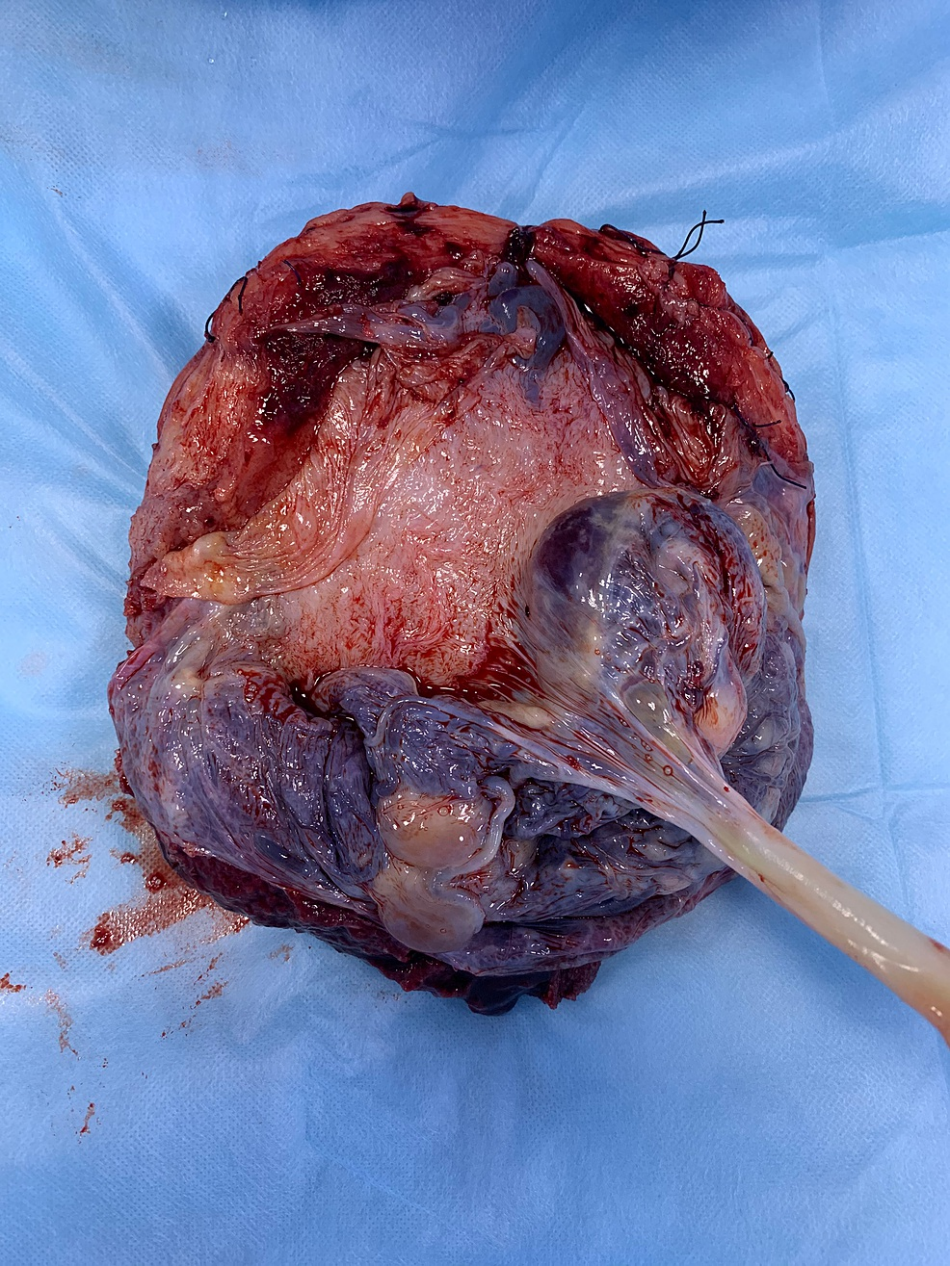
Case 2 showing placenta increta

**Figure 2:**
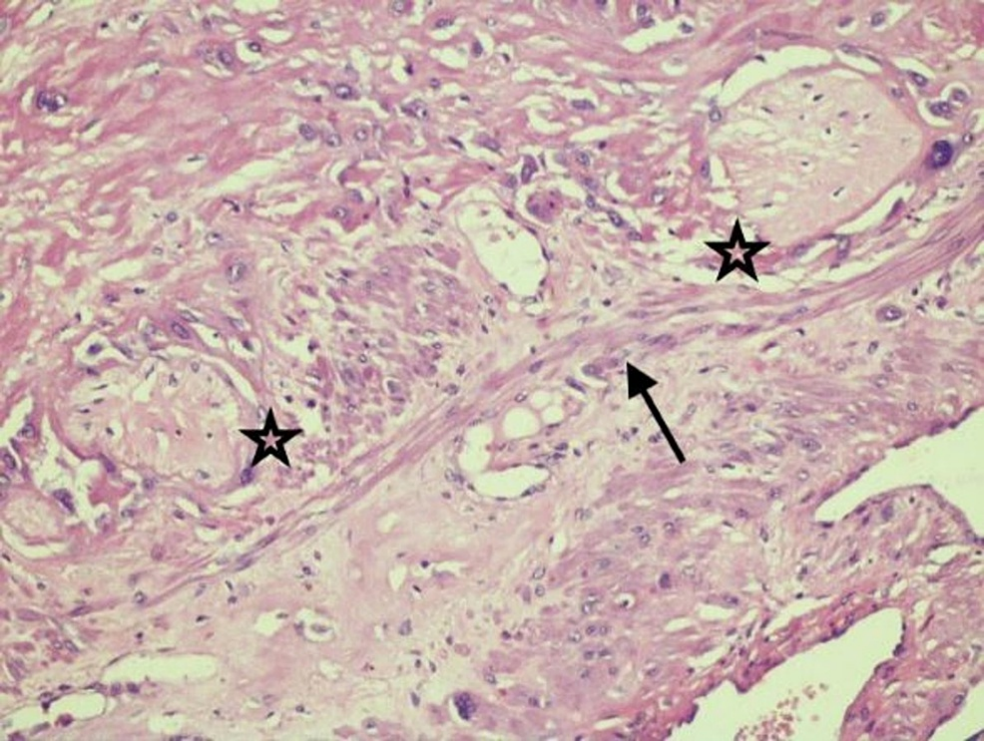
Microphotograph of placenta increta showing hyalinised chorionic villi (star) extended into the muscle bundles (arrow) of myometrial tissue (Hematoxylin and Eosin X200)

**Figure 3:**
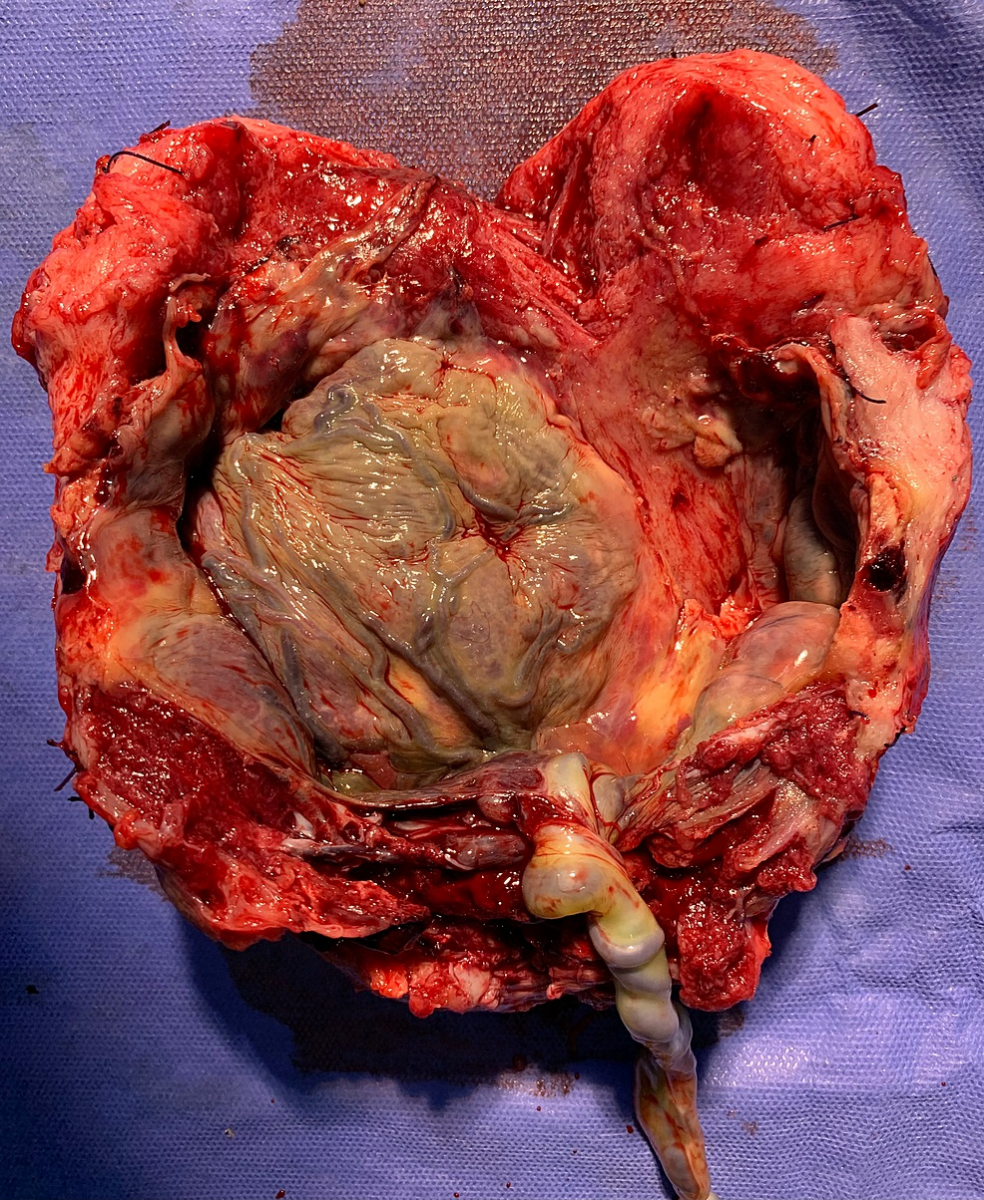
Case 3 showing placenta increta

**Figure 4:**
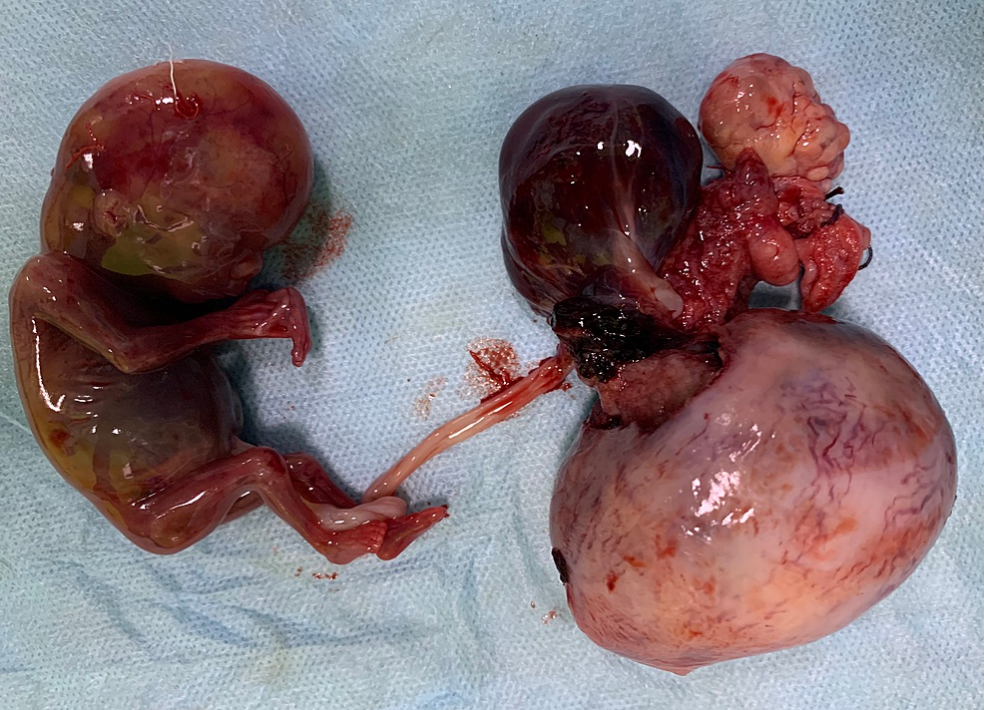
Case 7 showing ruptured rudimentary horn pregnancy

**Figure 5:**
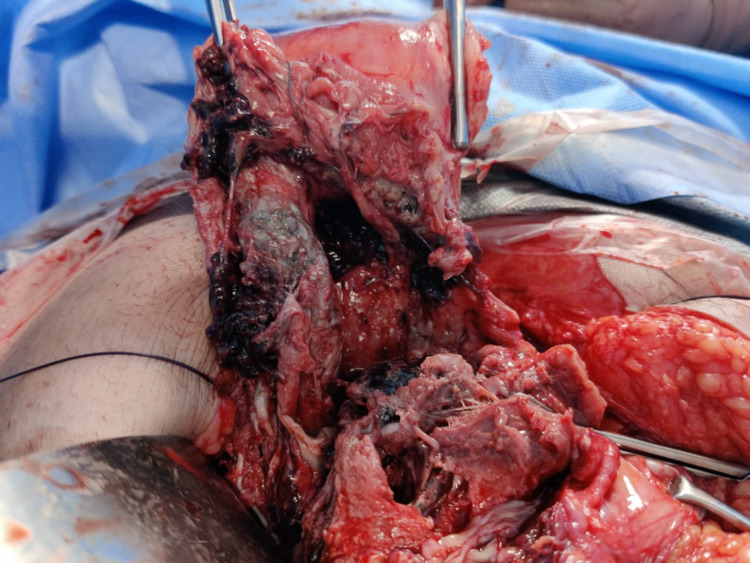
Case 8 showing necrotic placental tissue

Table 2 shows the intraoperative and postoperative findings of EPH cases. Case number 1-3 had central placenta praevia (Figures 1-3). Case 4 had a massive postpartum haemorrhage and bleeding continued despite stepwise devascularisation. In the 5th case, the foetal head was deeply impacted in the pelvis and had extension of uterine incision into broad ligaments and inferiorly into the vagina. Despite best possible efforts to repair the extensions with step-wise devascularisation, bleeding continued resulting in a need for hysterectomy. The sixth case presented with frank haematuria with decreasing haemoglobin (9 to 6 gm%) levels and a history of on and off bleeding per vaginum. Cystoscopy showed bladder filled with clots but complete removal of clots was not possible due to large clots. Placental infiltration was also seen. Case 7 presented in haemorrhagic shock with ruptured rudimentary horn pregnancy (Figure 4). Case 8 and 9 had massive postpartum bleeding with gaped caesarean scar and atonic uterus respectively (Figure 5).

**Table 2:** Intraoperative and postoperative findings EPH: Emergency peripartum hysterectomy; APH: Antepartum hysterectomy; PPH: Postpartum hysterectomy; HPE: Histopathological examination.

S. no	Indication of EPH	Type of uterine incision in EPH	Intra-op findings
1	Massive PPH with placenta percreta	Lower segment	Central placenta praevia with percreta present, invading through serosa on the left lateral surface of the uterus; Bladder injury due to densely adhered bladder with uterus; Subtotal hysterectomy done. HPE: Placenta percreta
2	Placenta praevia with percreta	Transfundal	Bladder densely adhered to lower uterine segment with tortuous vessels. Placenta bulging through lower uterine segment and seen covered with peritoneum (Figure 1). HPE: Placenta increta (Figure 2)
3	Placenta praevia with percreta with APH	Transfundal	Bladder pulled up and completely adhered to the anterior wall of the uterus, placenta seemed involving posterior wall of the urinary bladder. The bladder was injured during dissection (Figure 3). HPE: placenta increta
4	PPH	Transfundal	Bilobed placenta removed; Increta suspected, placental bed bleeding despite stepwise devascularisation. HPE: Placenta was normal with no invasion.
5	Traumatic PPH	Lower segment	Head deep in the pelvis; Traumatic postpartum haemorrhage noted. (Extension was present bilaterally into both broad ligaments & extension inferiorly up to vagina; Repair of extensions attempted but the patient condition deteriorated with continuous bleeding. HPE: Placenta was normal with no invasion.
6	Frank haematuria with decreasing haemoglobin (9 to 6 gm%), bleeding on and off	Lower segment	Cystoscopy done, bladder filled with clots, complete removal of clots not possible due to large clots. Placental infiltration seen. Bilateral ureteric orifice normal. Hysterotomy done. Torrential bleeding noted from the lower uterine segment for which subtotal hysterectomy was done. Intentional cystotomy f/b bladder clot evacuation. HPE: Placenta percreta
7	Ruptured rudimentary horn pregnancy with haemorrhagic shock	Hemi-hysterectomy	3 litres (60% blood loss) of blood and blood clot removed, rupture in communicating rudimentary horn of uterus; Fetus was seen lying in peritoneal cavity attached with placenta via umbilical cord; Resection of left horn of uterus done along with placenta in situ (Figure 4). HPE: placenta increta in rudimentary horn
8	Secondary PPH with puerperal sepsis	-	Previous scar gaped away, placenta coming out of scar site; Necrotic tissue seen over the lower uterine segment (Figure 5). HPE: Placenta was normal with no invasion.
9	PPH	-	4x4 cm episiotomy site haematoma, bleeding actively -drained; Uterus flabby; Step-wise devascularisation done- uterus still flabby- subtotal hysterectomy done. HPE: Placenta was normal with no invasion.

Table 3 shows the intraoperative blood loss, intraoperative transfusion, postoperative transfusion, maternal intensive care unit stay and major concerns of the mother once she stabilised. As seen in Table 3, all women required multiple blood component transfusion both intra- and post-operatively. Five out of nine women (55.56%) needed ICU care. Once stabilised, their major concerns were related to their newborn child.

**Table 3:** Maternal morbidity and concerns Intraop: Intraoperative; Postop: Postoperative; ICU: Intensive care unit; PRBC: Packed red blood cell; FFP: Fresh Frozen Plasma; Cryo: Cryoprecipitate; RDP: Random Donor Platelets; WB: Whole blood.

S. no.	Blood loss	Intraop transfusion	Postop transfusion	Maternal ICU stay	Number of days on ventilator	Concerns of mother
1	5 L	5 PRBC + 2 WB + 4 Plt + 4 FFP + 4 cryo	2 PRBC	3	3	Fear about the health of COVID positive husband
2	2.5 L	1 PRBC + 2 FFP	5 PRBC, 4 FFP, and 4 RDP	None	-	Newborn baby
3	2 L	2 PRBC + 4 RDP	3 PRBC + 4 FFP + 4 RDP	None	-	Newborn baby
4	2.5 L	3 PRBC + 3 FFP + 3 RDP	-	None	-	Worried about husband's COVID status (Husband turned positive)
5	2.5 L	4 PRBC + 4 FFP + 4 platelets + 2 cryoprecipitate	1 PRBC + 6 cryoprecipitate	4 days; Ventilatory support – 2 days, followed by metabolic acidosis correction	2	Worried about the baby as she knew about meconium staining
6	5 L	12 crystalloids, 8 PRBC, 7 RDP, 6 FFP	10 units Cryoprecipitate + 3 units PRBC + 2 units FFP + 4 RDP	2 days	2	Worried about her kids at home
7	3 L	4 unit PRBC, 4 unit RDP and 4 unit FFP	1 PRBC	2 days	1	None
8	1 L	2 PRBC + 4 unit FFP	-	None	-	Worried about her kids at home
9	2 L	2 PRBC + 4 FFP + 4 platelets	4 PRBC + 4 FFP + 4 platelets	3 days	2	Worried about her newborn baby
	Mean loss 2.83 ± 1.34					

Table 4 shows the infant characteristics with the period of separation from the mother. Seven out of nine women (77.78%) had live births and two of these infants died.

**Table 4:** Infant characteristics NICU: Neonatal Intensive Care Unit

S. no.	Live birth (yes/no)	Birth weight	NICU stay Yes/no	Period of separation from mother in days
1	Yes	2.490	Yes	4 days
2	Yes	2.2	Yes	1 day
3	Yes	950 gm	Died within half an hour of birth, congenitally malformed baby	Baby died
4	Yes	3.2	Yes	1 day
5	Yes	2.19	Yes	5 days
6	No	620 gm initial resuscitation attempted but no signs of respiration noted	none	Baby died
7	None	Abortion	none	abortion
8	Yes	1.8 kg	yes	2 days
9	Yes	2.8 kg	Yes, on ventilator, expired on day 6 in view of severe birth asphyxia	Baby finally expired.

As seen in Table 5, all these women reported some health issues post-hysterectomy during their six-week follow-up. The guilt of losing the baby, lethargy, worries related to feminity and sexual health, and flashbacks of ICU stay were significant concerns.

**Table 5:** Self-reported health problems associated with traumatic birth at 6 weeks visit

S. no.	Problems
1	Physically exhausted, Reluctant to feed the baby and not emotionally attached to the newborn child
2	Worried about loss of feminity
3	Unable to get over the loss of her newborn baby, loss of feminity
4	Lethargy, unable to take care of her children
5	Worried about feminity, feeling weak
6	Guilt of losing baby
7	Flashbacks of ICU stay and fear of death, emotionally labile, feeling weak
8	Worried about future sexual life
9	Flashbacks of ICU stay and fear of death, guilt of losing baby, feeling weak

## Discussion

Emergency peripartum hysterectomy (EPH) is an obstetric procedure performed in a crisis as last-resort, life-saving surgery, typically following intractable postpartum hemorrhage [13,14]. EPH is classified as severe maternal morbidity, or even near-miss maternal mortality, due to the surgical intervention, intubation, blood transfusions, and critical care transfer associated with it [15]. The purpose of this study was to analyse women undergoing EPH during the COVID pandemic in terms of their sociodemographic features, indications, intraoperative and postoperative complications, neonatal parameters, concerns, and worries related to traumatic childbirth. To date, no studies focussing on the demographic details and causes of emergency peripartum hysterectomy during the COVID-19 pandemic are recorded in the literature.

The memories of childbirth last a lifetime, and every woman cherishes and discusses them with her friends and family members. A bad experience can make her lose confidence in the health system. This pandemic has created a sense of insecurity with concerns of vertical transmission in obstetric patients. Pregnant women have become extremely cautious in seeking health facilities to avoid being getting exposed unless deemed necessary. Lockdown travel restrictions have resulted in women avoiding routine check-ups. Despite centres running telemedicine facilities, many high-risk factors get missed due to a lack of physically examining pregnant women. Such women usually present in labor with high-risk factors, often in subcentres or similar hospitals wherein there is a shortage of skilled healthcare workers, requiring referrals to higher facilities.

Goyal et al. [16] evaluated their experience of the COVID-19 pandemic on maternal health due to delay in seeking health care. They found that around 32.5% of pregnant women had fewer antenatal visits during the pandemic. The primary reasons for the delay in seeking help were quoted as strict lockdown resulting in lack of transportation facilities (50.9%) and fear of getting COVID infection in 33.4%. In our study, all women were unbooked, with 77.78% (seven out of nine) referred to our centre with high-risk factors. The primary indication of EPH in our study sample was obstetric haemorrhage due to morbidly adherent placenta. A transfundal uterine incision, when feasible, was preferred compared to a lower segment incision in emergencies wherein the diagnosis of morbidly adherent placenta was made preoperatively. Five women had morbidly adhered placenta and had massive blood loss during caesarean section necessitating emergency hysterectomy despite step-wise systematic devascularisation. No definite guidelines exist for the step at which hysterectomy becomes mandatory, and this is primarily a subjective decision. In a 26-year review of around 56 cases of EPH from a tertiary centre in Kuwait, the authors found that almost 37% of cases had at least one previous caesarean section [17]. In our cohort of EPH cases, we had 66.67% cases with a previously scarred uterus. With rising caesarean section rates, there is a definite rise in incidences of the adhered placenta, leading to an increase in cases of EPH [18]. Though this can be prevented if diagnosed early due to advancements in early detection by doppler sonography in conjunction with magnetic resonance imaging, hysterectomy is often the only resort when a patient presents in labour.

EPH is associated with massive blood loss. The mean blood loss in our cohort was 2.83 ± 1.34 as compared to 3467 ± 2110 in a study by Chibber et al. [17]. Five women required intensive care unit (ICU) support owing to massive blood loss. None of the women died in our study, unlike two maternal deaths in the study by Chibber et al. [17]. Neonatal outcome was poor in our study sample, with two early neonatal deaths amongst seven live births.

These women were asked to mention their significant concerns after stabilisation and discharge from ICU. The primary concerns were mostly related to the health of the newborn child and husband. Zanardo et al. suggested pregnant women delivering during this COVID-19 pandemic as a vulnerable group need careful follow-up to improve psychological wellbeing [19]. Traumatic childbirth may further aggravate the emotional health of such women. In our cohort of nine cases of EPH, problems related to physical exertion, loss of feminity, and guilt of losing a child were the main issues. Health care providers often miss this aspect of postpartum care as women hardly speak about their emotional health, especially in rural areas. By identifying the experience and sequelae of EPH, health care providers can better understand the concerns of these women and add to women’s satisfaction and mental health. Here lies the importance and need for a preventive psychological session for a woman with a traumatic birth.

A major strength of this study is its unique nature in identifying the sociodemographic risk factors of EPH during the COVID pandemic and addressing the self-reported health issues on the postpartum visit. The study could have been better if we had used some standard questionnaire to address post-traumatic stress disorder and depression and identify those at risk for stress and depression, but being a retrospective study, we lagged in this aspect. Future research is needed to address the gap in knowledge on EPH in terms of experience and its sequelae.

## Conclusions

The morbidly adhered placenta was the primary cause of EPH in our study cohort. In our study cohort, all women were unbooked and 77.78% (seven out of nine) were referred to our centre with high-risk factors. Here lies the importance of antenatal care for timely identification of high-risk cases so that antenatal counselling can be done. Prompt resuscitation with early surgical intervention in a well-equipped tertiary centre probably reduced the morbidity and prevented maternal death.

The preventive psychological session should be part of postpartum follow-up visits for any women with traumatic childbirth. Just making women survive from a near-miss event does not add to successful clinical outcome as many of these women may suffer in silence and agony owing to traumatic childbirth. By improving our understanding of postpartum needs, we can deal with the unmet mental health concerns of the women undergoing emergency obstetric hysterectomy.
